# Assessing the Nutritional Knowledge Gap: Students’ View of UK Medical School Education

**DOI:** 10.7759/cureus.96280

**Published:** 2025-11-07

**Authors:** Diya Sethi, Seema Sethi

**Affiliations:** 1 Medicine, University of Buckingham, Crewe, GBR; 2 General Practice, NHS Wales, Newport, GBR

**Keywords:** diet, medical curriculum, nutrition, nutrition education, uk medical schools, undergraduate medical education

## Abstract

This study aimed to examine medical students’ views on nutrition education in UK medical schools. It focuses on the adequacy of training, confidence in applying nutritional knowledge, and gaps in the current curriculum. An online survey, completed by 92 students from 17 medical schools, explored their views on the role of nutrition in healthcare, the quality of training received, their confidence in using this knowledge, and their preferred teaching methods. Of note, 87% of students believed doctors should provide nutritional advice. However, only 19.6% felt confident conducting nutritional assessments, and 49% lacked confidence in offering dietary guidance. Over half (56%) of the students reported receiving less than four hours of nutrition training. Many relied on external resources, especially websites, to fill knowledge gaps. These findings highlight the need to improve nutrition education in medical schools. Recommended approaches include embedding nutrition topics into clinical modules, using active learning methods like case-based learning, and offering practical sessions led by dietitians. Reforming the curriculum is crucial to equip future doctors with the skills to manage nutrition-related conditions and promote public health.

## Introduction

Globally, conditions such as ischemic heart disease, stroke, lung cancer, and diabetes were among the top 10 causes of death in 2021. Key risk factors for these diseases include high blood pressure, elevated low-density lipoprotein (LDL) cholesterol, low birth weight, and high plasma glucose levels. These risk factors are projected to continue shaping the global disease burden through 2050 [1]. In the UK alone, 75,000 premature deaths are associated with unhealthy diets each year, including almost 17,000 deaths among individuals aged 15-70 years [2]. Nutrition plays a critical role in the development and management of these conditions, highlighting the need for healthcare professionals to be adequately trained in this domain [3-5].

Despite the clear significance of nutrition in healthcare, doctors report feeling ill-prepared to provide evidence-based dietary guidance to their patients [6]. The integration of nutrition teaching remains inconsistent across medical schools, with significant variation in whether it is incorporated into the core curriculum or offered only as an elective [7]. While previous studies have examined attitudes toward nutrition education, confidence levels, and exposure to training at individual institution levels, there is still a lack of comprehensive, targeted, up-to-date evaluations focused on the UK medical schools as a whole, with a primary focus on students [6,8,9]. Hence, this study aims to evaluate medical students’ perceptions of nutrition education in UK medical schools, assess their confidence in understanding nutritional concepts, and identify gaps and preferred learning methods to inform the enhancement of nutrition education in the medical curriculum.

## Materials and methods

An online survey comprising 12 questions was developed using Microsoft Forms to gather data from medical students across the UK. The survey was structured to include three main components. First, it collected demographic information such as medical school affiliation, year of study, and expected year of graduation through closed-ended questions. Second, it incorporated Likert-scale items to assess students’ perceptions regarding the importance of nutrition in healthcare, the adequacy of nutrition education within their curriculum, and their confidence in providing dietary advice. Third, multiple-choice questions were used to explore the types of external resources students accessed and to quantify the number of hours of formal nutrition education they had received. To minimise response bias, the survey was carefully worded using neutral language. Standardised Likert scales and multiple-choice formats were employed to avoid leading participants toward specific responses.

This cross-sectional study specifically targeted students who had substantial exposure to the medical curriculum. For undergraduate programmes spanning five years, students in Years four and five were invited to participate. Graduate-entry courses, typically four years in duration, included students from Years three and four. In six-year programmes, the survey was directed at those in Years five and six. Exclusion criteria were applied to ensure relevance and consistency in the sample. Students who were not enrolled in a UK medical school, those who had not yet entered clinical or placement years, and individuals studying allied health courses that did not lead to a medical degree or qualification as a doctor were excluded from participation.

Ethical approval for the study was initially granted by the University of Buckingham and the Medical Schools Council. Following this, Programme Directors at various medical schools were contacted with a request to distribute the survey. Data collection was conducted between July and September 2024. Participants were recruited using a non-probability convenience sampling technique, targeting eligible medical students from UK medical schools who were accessible via education leads and willing to participate during the survey period. No formal sample size calculation was performed, as the study was exploratory in nature. The sample size was determined pragmatically based on anticipated response rates and recruitment feasibility during the survey period. Data analysis was performed using SPSS Statistics version 29.0 (IBM Corp., Armonk, NY). Descriptive statistics, including percentages, were calculated, and chi-square tests were applied where appropriate to examine associations between variables.

Participation in the survey was entirely voluntary, and anonymity was assured throughout. All responses were securely stored on the university’s database, with access restricted to the lead researcher. Informed consent was obtained at the beginning of the survey.

## Results

Response overview

A total of 132 survey responses were collected from 17 medical schools across the UK. Following the exclusion of 40 responses that did not meet the inclusion criteria based on the year group, 92 responses were included for analysis.

Demographics

The majority of responses were from fourth-year medical students (38 (41%)), followed by fifth-year students (27 (29%)). Among the institutions, the University of Buckingham recorded the highest response rate (22 (24%)), followed by Plymouth (15 (16%)), Oxford (11 (12%)), and Ulster (9 (9%)) (Table 1).

**Table 1:** Distribution of student survey respondents by medical school and year of study (responses from non-clinical years not included)

Medical school	Year of study	Number of respondents
University of Buckingham	3	10
University of Buckingham	4	10
University of Buckingham	5	1
University of Buckingham	6	1
University of Oxford	5	7
University of Oxford	6	4
University of Plymouth	4	6
University of Plymouth	5	4
Ulster University	3	4
Ulster University	4	5
King's College London	5	2
University of Birmingham	4	3
University of Birmingham	5	3
University of Cambridge	3	1
University of Cambridge	5	2
University of Cambridge	6	1
University of Glasgow	4	2
University of Glasgow	5	1
Peninsula Medical School	4	3
Peninsula Medical School	5	2
Norwich Medical School	4	4
Norwich Medical School	5	1
Other institutions	3–5	≤2 each

Perceived importance of nutrition in medical practice

**Figure 1:**
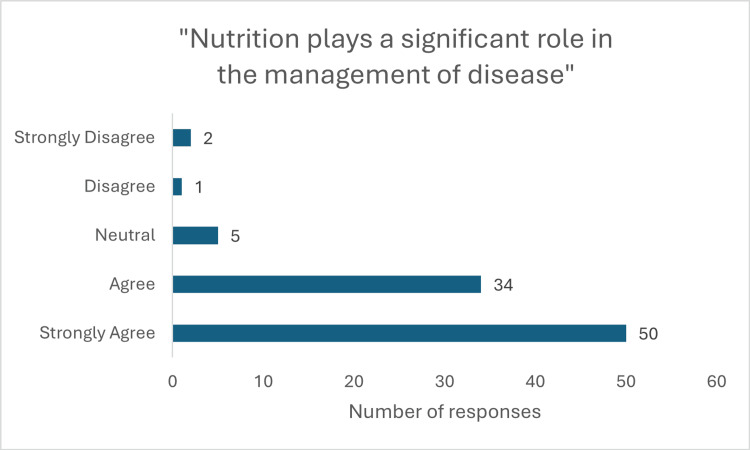
Response to question "Nutrition plays a significant role in management of disease"

**Figure 2:**
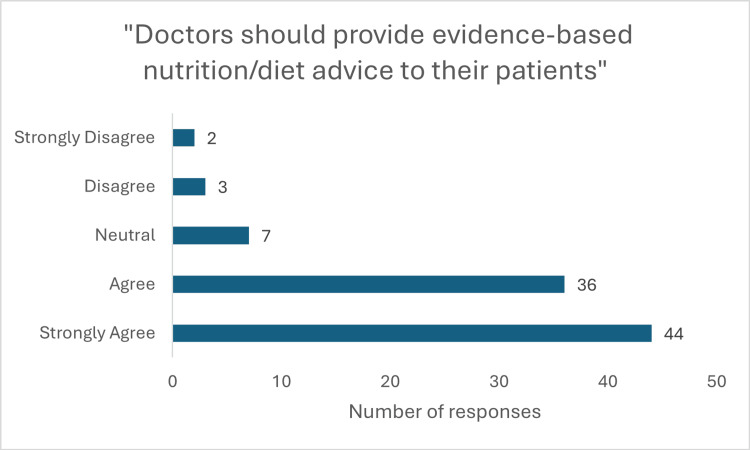
Response to question "Doctors should provide evidence-based nutrition/diet advice to their patients"

The survey revealed a strong consensus among students regarding the significance of nutrition in clinical practice. A majority agreed that nutrition is crucial in the management of disease (50 (54.3%) strongly agree, 34 (37%) agree; p<0.001, x^2^=108.33) and an even greater proportion acknowledged its role in disease prevention (63 (68.5%) strongly agree, 25 (27.2%) agree; p<0.001, x^2^=108.09)(Figure 1). This underscores the vital role nutrition plays in preventing chronic conditions. Additionally, 80 (87%) respondents (44 (47.8%) strongly agree, 36 (39.1%) agree; p<0.001, x^2^=87.02) believed it is a doctor’s responsibility to provide nutritional advice (Figure 2), while 73 (80%) (34 (37%) strongly agree, 39 (42.4%) agree; p<0.001, x^2^=63.65) felt patients expect such guidance from their physicians.

Adequacy of nutrition education in the medical curriculum

**Figure 3:**
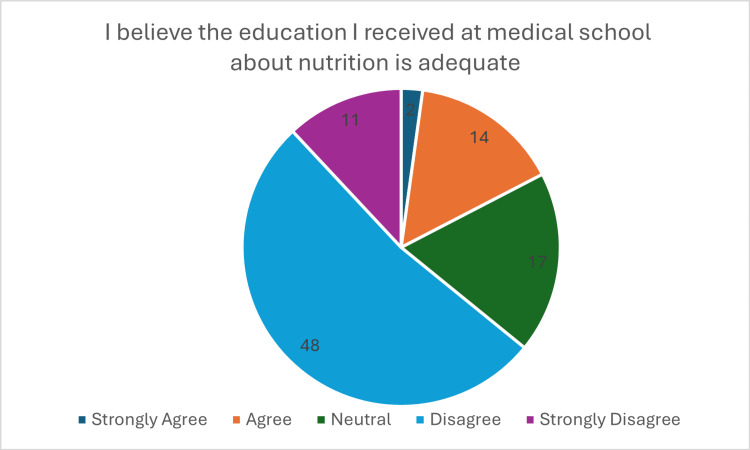
Response to question "I believe the education I received at medical school about nutrition is adequate"

Despite widespread recognition of nutrition’s importance, significant gaps in medical education were evident. While 65 (71%) respondents reported receiving some form of nutrition teaching, 56% noted this amounted to fewer than four hours. Furthermore, 59 (78.3%) (p <0.001, x^2^=66.37) considered the current nutrition education within medical school curricula to be inadequate (Figure 3).

Confidence in applying nutritional knowledge

**Figure 4:**
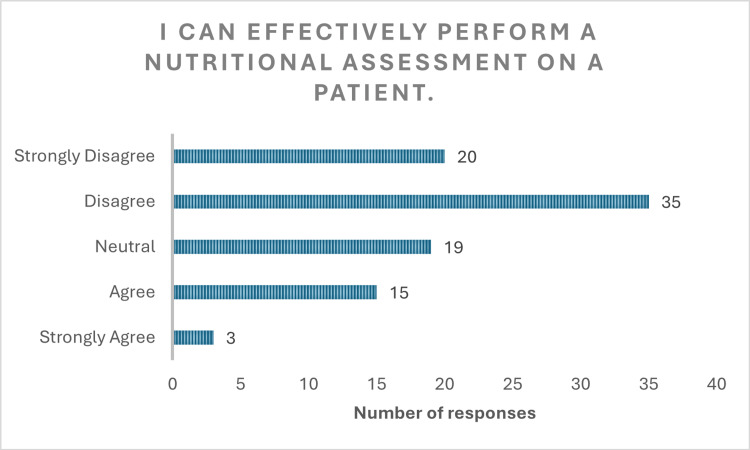
Response to question "I can effectively perform a nutritional assessment on patient"

**Figure 5:**
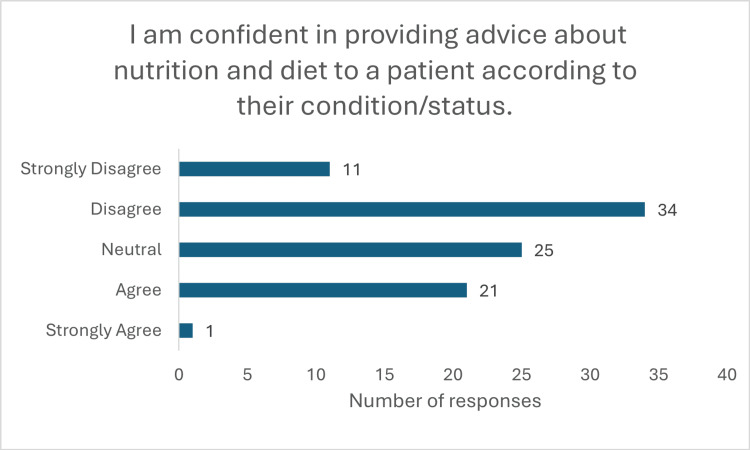
Response to question "I am confident in providing advice about nutrition and diet to a patient according to their condition/status"

**Figure 6:**
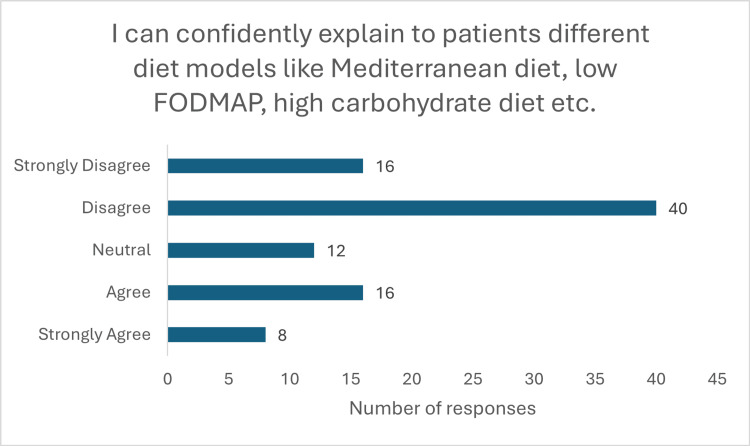
Response to question "I can confidently explain to patients different diet models like Mediterranean diet, low FODMAP, high carbohydrate diet, etc."

**Figure 7:**
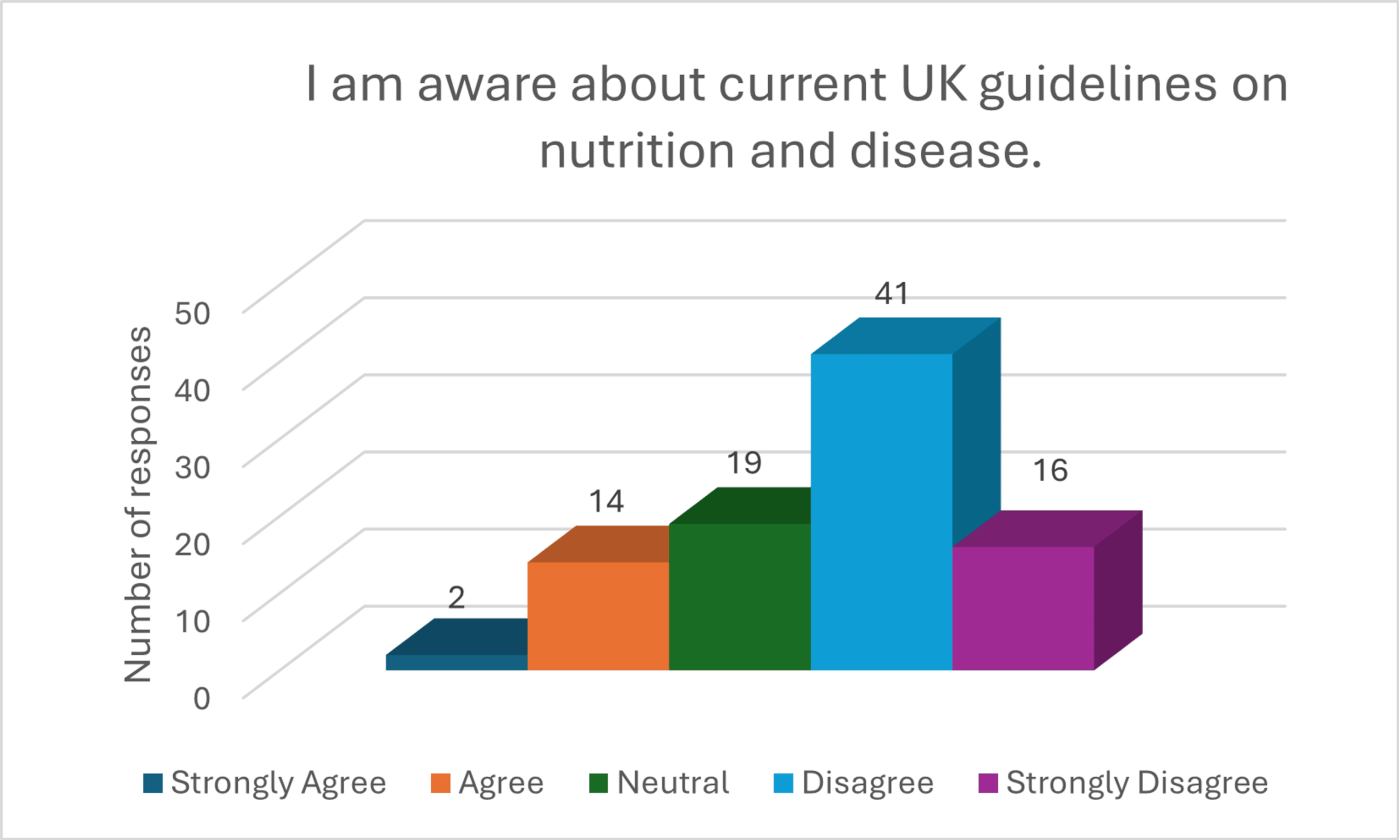
Response to question "I am aware about current UK guidelines on nutrition and disease"

The findings also highlighted a lack of confidence among medical students in applying nutritional knowledge in clinical settings. Only 29 (39.1%) (p<0.001, x^2^=44.40) respondents expressed confidence in their understanding of nutrition, with 33 (36%) remaining neutral. Alarmingly, only 18 (19.6%) (p<0.001, x^2^=28.66) felt capable of conducting a nutritional assessment, while 54 (59.7%) reported they could not (Figure 4). Nearly half (45 (49%)) were not confident in offering dietary advice to patients, and 25 (27.2%) remained neutral on the matter (Figure 5) (p<0.001, x^2^=35.40). Furthermore, 56 (60%) students admitted unfamiliarity with diet models such as the FODMAP, Mediterranean, and low-residue diets. Only 16 (19%) (p<0.001, x^2^=43.76) were aware of the UK’s nutrition and disease guidelines (Figures 6, 7).

Interest in expanded nutrition education

**Figure 8:**
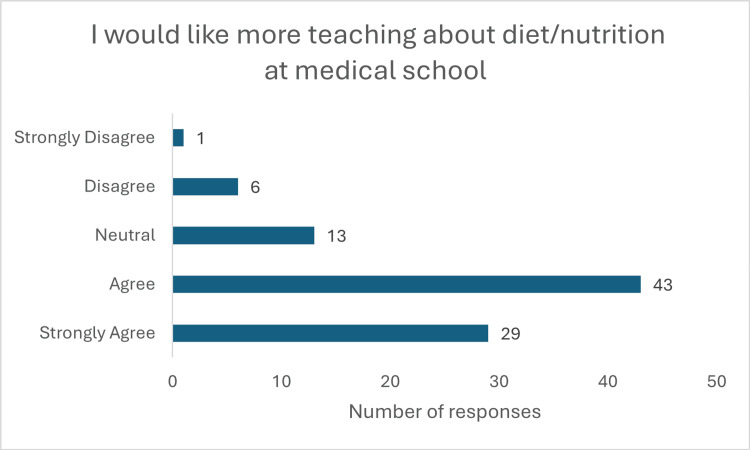
Response to question "I would like more teaching about diet/nutrition at medical school"

**Figure 9:**
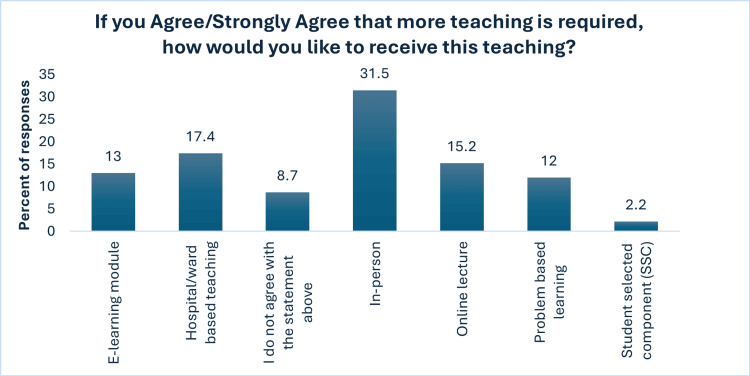
Response to question "If you Agree/Strongly Agree that more teaching is required, how would you like to receive this teaching?"

The survey demonstrated a strong interest among students in expanding their nutritional education, with 72 (78.3%) expressing a desire for more comprehensive training (Figure 8). Among preferred teaching methods, in-person and hospital-based instruction emerged as the most favoured (Figure 9).

Supplementary learning resources

To address the gaps in their formal education, many students sought external resources, with 71 (77.2%) utilising materials not provided by their universities. Websites were the most accessed supplementary resource.

## Discussion

This study highlights critical gaps in nutrition education within UK medical schools, despite strong student recognition of its importance in disease prevention and management. Our survey found that 87% of respondents believe doctors should provide nutritional advice, and 80% think patients expect such guidance. However, only 19.6% felt capable of conducting a nutritional assessment, and nearly half (49%) lacked confidence in offering dietary advice. These findings echo a long-standing global concern about the inadequacy of nutrition education in medical training, as documented in studies from Canada, Australia, and various UK institutions with a theme of dissatisfaction with the curriculum and low confidence in providing nutrition advice [7-10,11].

The shortfall in formal nutrition teaching is evident in the reported low contact hours: 56% of students indicated receiving fewer than four hours of training, as well as limited familiarity with standard diet models and poor awareness of UK nutrition guidelines (only 19% reported confidence in this area). As a result, students frequently turn to external sources, particularly websites, to fill knowledge gaps. Similar patterns identified at Brighton and Sussex Medical School prompted targeted interventions, including the recruitment of nutrition-trained staff and expansion of curriculum objectives [6]. These initiatives led to increased student interest and greater engagement with nutrition topics [7].

At a national level, frameworks such as the General Medical Council’s (GMC) Outcomes for Graduates and competency-based models in Australia provide a foundation for curriculum development in medical nutrition education, along with commitment to better implementation in the NHS Long Term Plan [12-14]. The Association for Nutrition’s (AfN) UK Undergraduate Curriculum in Nutrition for Medical Doctors (2021) offers a valuable structure for integrating nutrition education across medical training. Developed through broad stakeholder engagement, the AfN curriculum demonstrates how nutrition content aligns not only with GMC outcomes but also with broader clinical areas, including gastroenterology, cardiopulmonary care, surgery, primary care, and public health [15]. Embedding nutrition education in clinical modules such as endocrinology and gastroenterology, and introducing practical skills - such as dietary history taking and weight management - early in medical training, has been shown to improve both knowledge and confidence [16,17].

Innovative teaching approaches such as active learning strategies - case-based learning, flipped classrooms, and practical workshops - have also shown promise in enhancing student engagement and competence [18,19]. Role modelling and emphasis on the relevance of nutrition have been identified as important factors in improving the gap [20]. For instance, dietitian-led sessions and culinary medicine programs have improved not only students’ clinical skills but also their own dietary behaviours [21,22].

Despite persistent challenges like curriculum overcrowding and a shortage of trained staff, scalable technological solutions - such as virtual simulations, interactive platforms, and access to curated digital resources - can help bridge the education gap [23]. Notably, the integration of nutrition need not require additional curriculum time. Successful models of vertical and horizontal integration embed nutrition content into existing modules, making learning more relevant and feasible within time-constrained programs [24]. These approaches, paired with interprofessional education and increased collaboration with nutrition experts, could reshape medical education to align with student and patient needs [25]. In addition, medical education should emphasise the importance of timely referrals to appropriately trained professionals, such as registered dietitians and diabetes educators, when specialised care is required.

Finally, this study underscores a strong student-led demand for more robust nutrition education and highlights its potential to enhance patient care. It is vital, however, to ensure appropriate signposting of high-quality nutrition resources, as reliance on media or personal beliefs may propagate misinformation. As shown in recent Australian studies, many students primarily use personal experience rather than evidence-based guidance to inform their nutritional recommendations [26]. Addressing these challenges through a multifaceted approach - curriculum integration, interprofessional collaboration, role modelling, and use of technology - will better equip future doctors to deliver competent, science-based nutrition care and ultimately improve health outcomes.

Limitations

This study is based on self-reported perceptions, which may not accurately reflect the actual nutrition content of medical curricula or the students’ competencies. The online survey tool was not pre-tested, which could have led to biases regarding the language of the questionnaire. No formal definition of “nutrition education,” “nutritional assessment,” or “UK nutrition and disease guidelines” was provided in the survey. As such, interpretations may have varied. Notably, students may have overlooked embedded or non-explicit content, such as disease pathophysiology, when estimating their exposure. The study did not verify student responses against curricular content or conduct formal assessments of nutritional knowledge.

The survey design did not gather information on the reliability of external sources that students used to supplement their knowledge. Additionally, we recognise that the inclusion of “high carbohydrate diet” may have led to confusion, as this term is not commonly emphasised in UK clinical nutrition. Discussions more often focus on low-carbohydrate diets, particularly in the context of diabetes and weight management. The survey could have been enhanced by including a free comment section to elaborate on the preferred form of teaching. Lastly, as participation was voluntary and promoted through education leads, there is a possibility of selection bias favouring students with pre-existing interest in nutrition. A bigger sample size would have improved the generalisability of the survey.

## Conclusions

This study highlights significant gaps in nutrition education within UK medical schools, despite strong recognition of its importance in disease prevention and management. Students reported low confidence in applying nutritional knowledge, limited formal training, and inadequate familiarity with diet models and guidelines, emphasising the need for curriculum reform and collaborative efforts with other healthcare professionals. The findings are limited by self-reported data, varying response rates across institutions, and the cross-sectional design, which precludes causal inferences. Future research should explore targeted interventions, large-scale studies improving on the limitations of this study, and scalable teaching methods to better prepare medical students for integrating nutrition into clinical practice.

## Appendices

Questionnaire

Academic Background

1. What year of medical school are you currently in?

Year 1

Year 2

Year 3

Year 4

Year 5

Year 6

2. Which medical school are you currently attending? (Open-ended response)

3. What is your expected year of graduation? (Open-ended response)

Role of Nutrition in Healthcare

4. Please tell me how strongly you agree or disagree with each one.

a. "Nutrition plays a significant role in the management of disease."

Strongly agree

Agree

Neutral

Disagree

Strongly disagree

b. "Nutrition can influence the development of a disease."

Strongly agree

Agree

Neutral

Disagree

Strongly disagree

Doctor’s Role in Nutrition

5. Again, please indicate how much you agree or disagree with the following statements.

a. "Doctors should provide evidence-based nutrition and diet advice to their patients."

Strongly agree

Agree

Neutral

Disagree

Strongly disagree

b. "Patients anticipate doctors to be capable of providing nutritional guidance."

Strongly agree

Agree

Neutral

Disagree

Strongly disagree

Nutrition Education in Medical School

6. Have you received any teaching about diet and nutrition at medical school?

Yes

No

7. Based on your university curriculum (excluding external resources), please rate the following statements.

a. "I believe the education I received at medical school about nutrition is adequate."

Strongly agree

Agree

Neutral

Disagree

Strongly disagree

b. "I would like more teaching about diet and nutrition at medical school."

Strongly agree

Agree

Neutral

Disagree

Strongly disagree

8. Approximately how many hours of teaching have you received about diet and nutrition in total?

0-2 hours

2-4 hours

4-6 hours

6-8 hours

More than 8 hours

9. If you agreed that more teaching is needed, how would you prefer to receive it?

Online lecture

In-person teaching

E-learning module

Problem-based learning

Hospital/ward-based teaching

Student Selected Component (SSC)

I do not agree that more teaching is needed

Confidence in Nutrition Knowledge

10. Please tell me how confident you feel about the following statements.

a. "I feel confident in my knowledge of diet and nutrition."

Strongly agree

Agree

Neutral

Disagree

Strongly disagree

b. "I can effectively perform a nutritional assessment on a patient."

Strongly agree

Agree

Neutral

Disagree

Strongly disagree

c. "I am confident in providing advice about nutrition and diet to a patient according to their condition or status."

Strongly agree

Agree

Neutral

Disagree

Strongly disagree

d. "I can confidently explain to patients different diet models like the Mediterranean diet, low FODMAP, high carbohydrate diet, etc."

Strongly agree

Agree

Neutral

Disagree

Strongly disagree

e. "I am aware of current UK guidelines on nutrition and disease."

Strongly agree

Agree

Neutral

Disagree

Strongly disagree

External Learning

11. Have you had to develop your knowledge about nutrition beyond the medical school curriculum using external resources?

Yes

No

12. If yes, what type of resources did you use?

Websites

Handbooks or textbooks

Hospital/ward teaching

Course or workshop

I selected ‘No’ in the previous question
